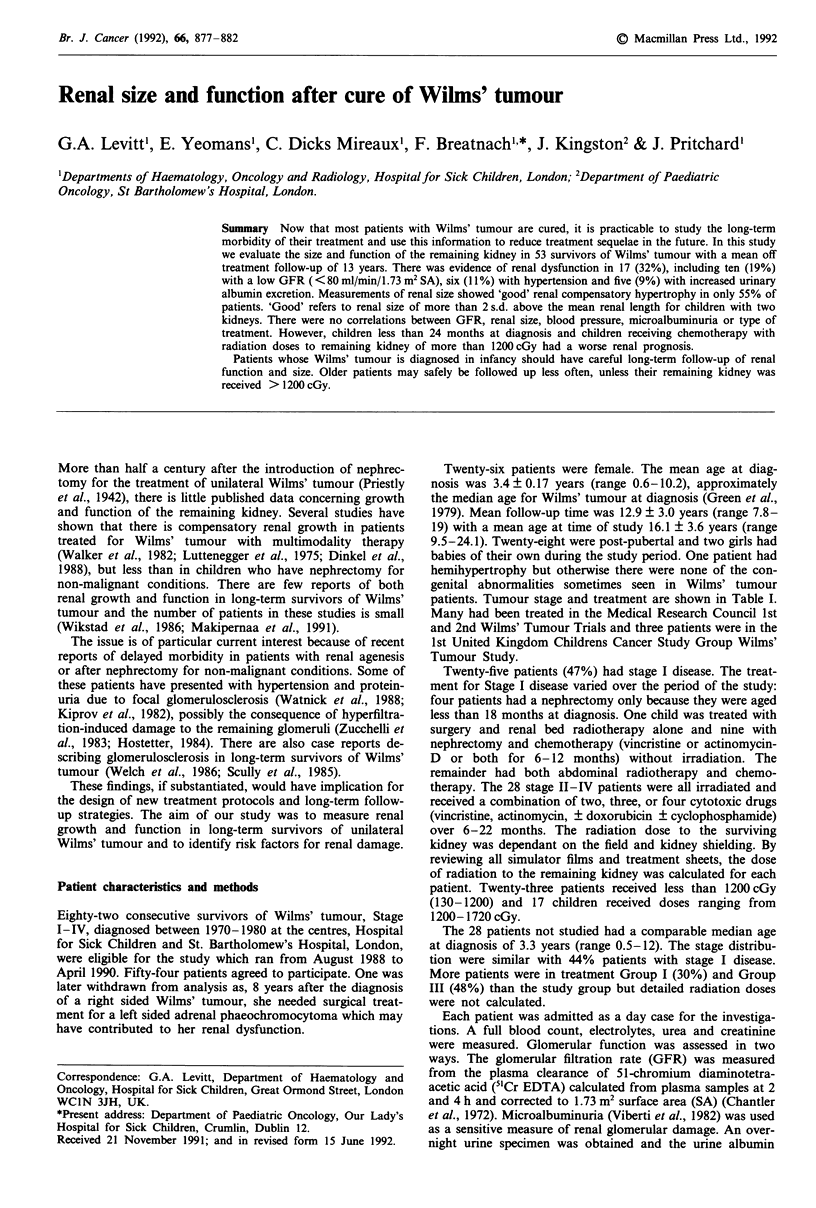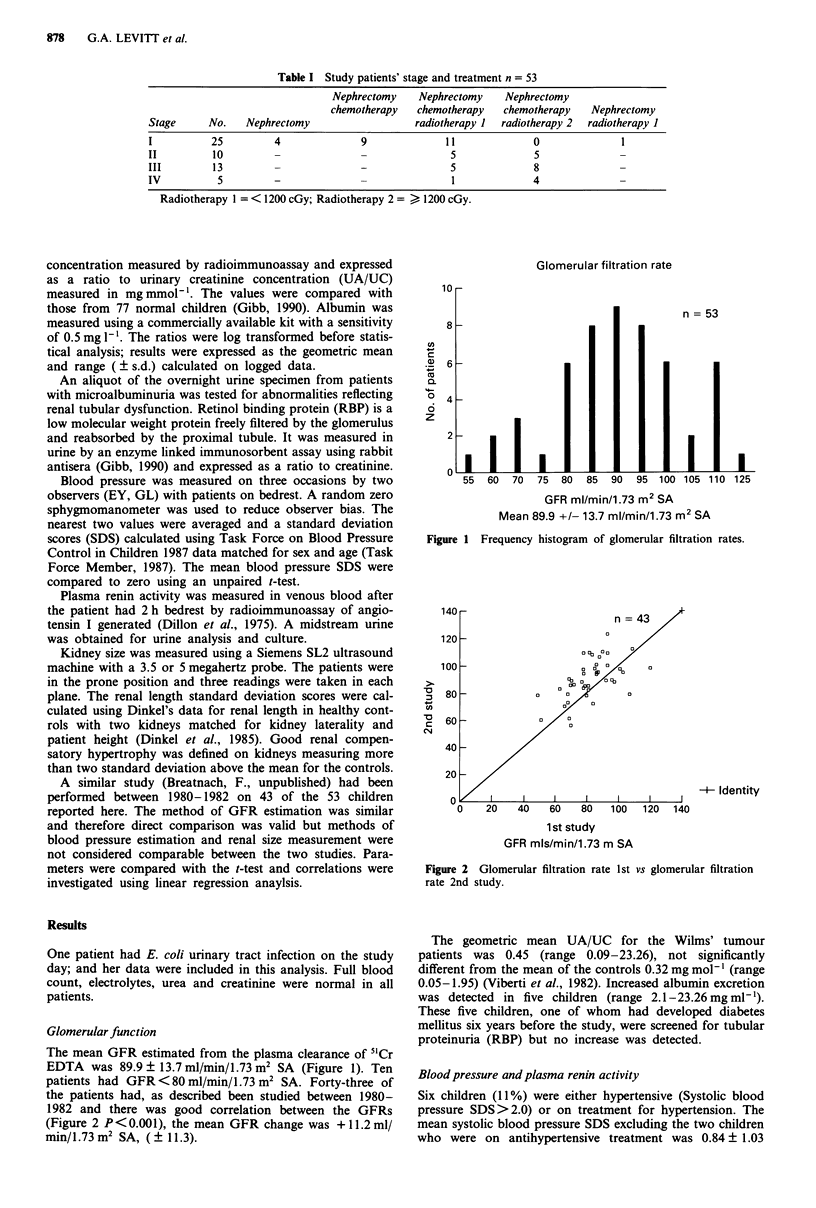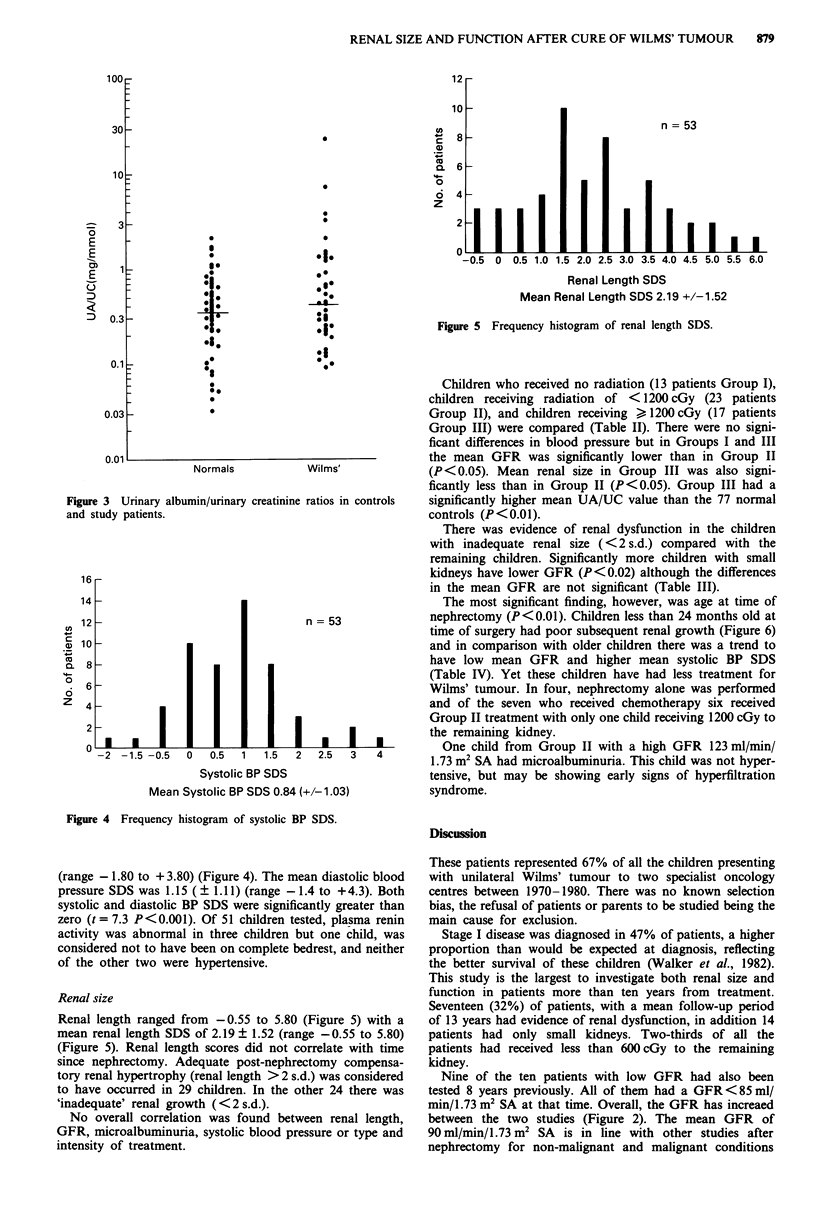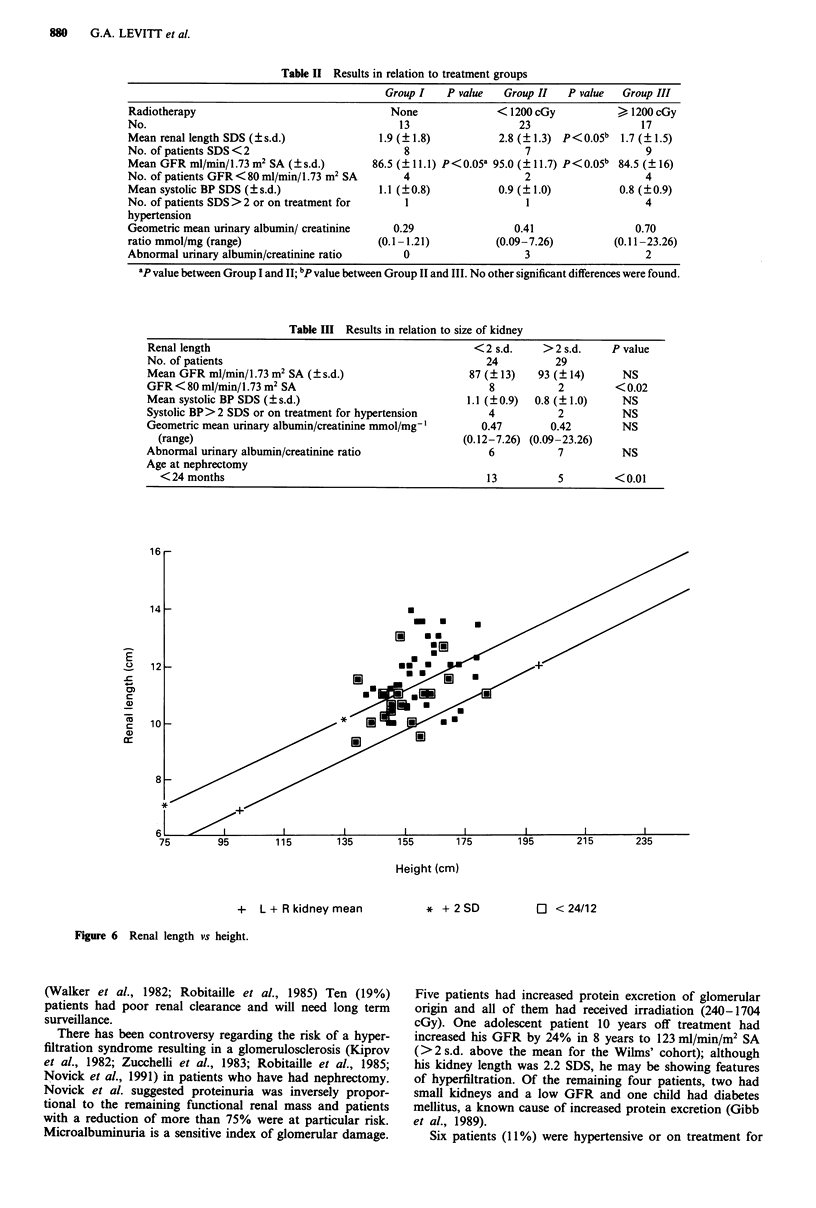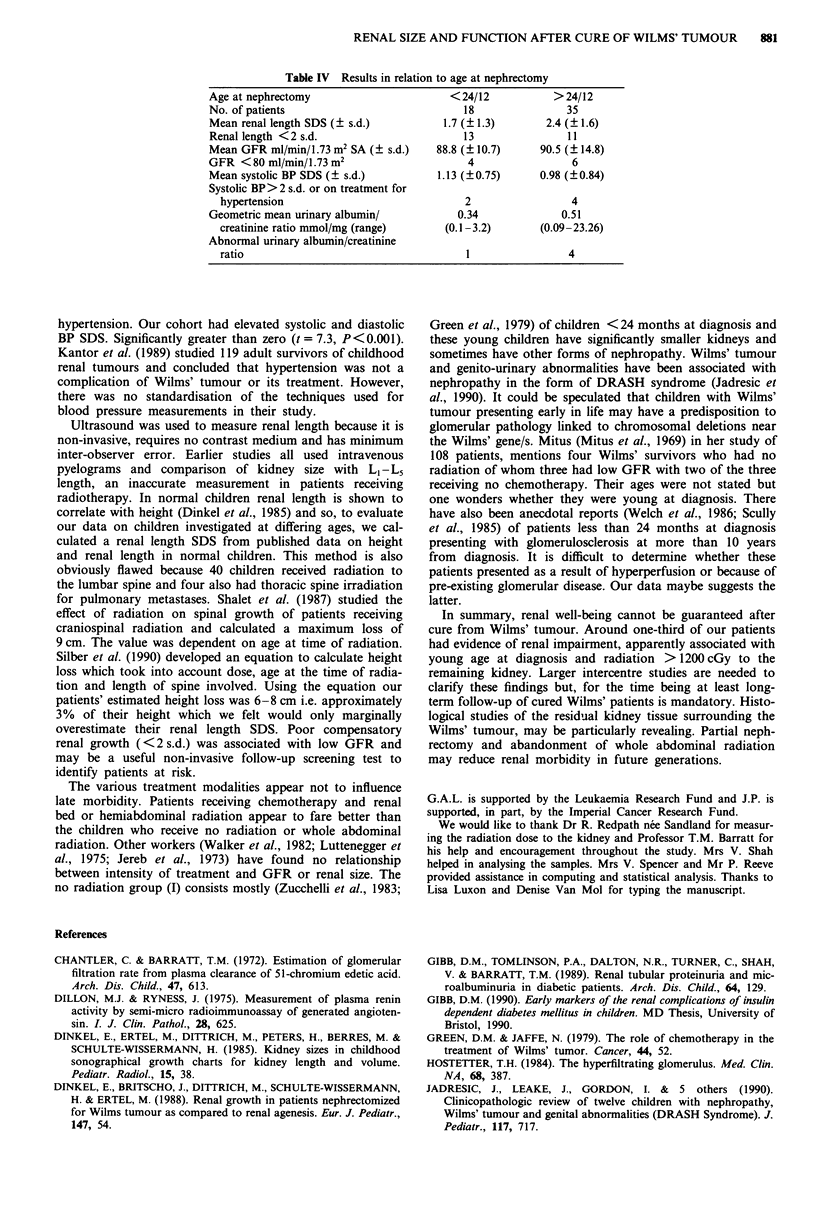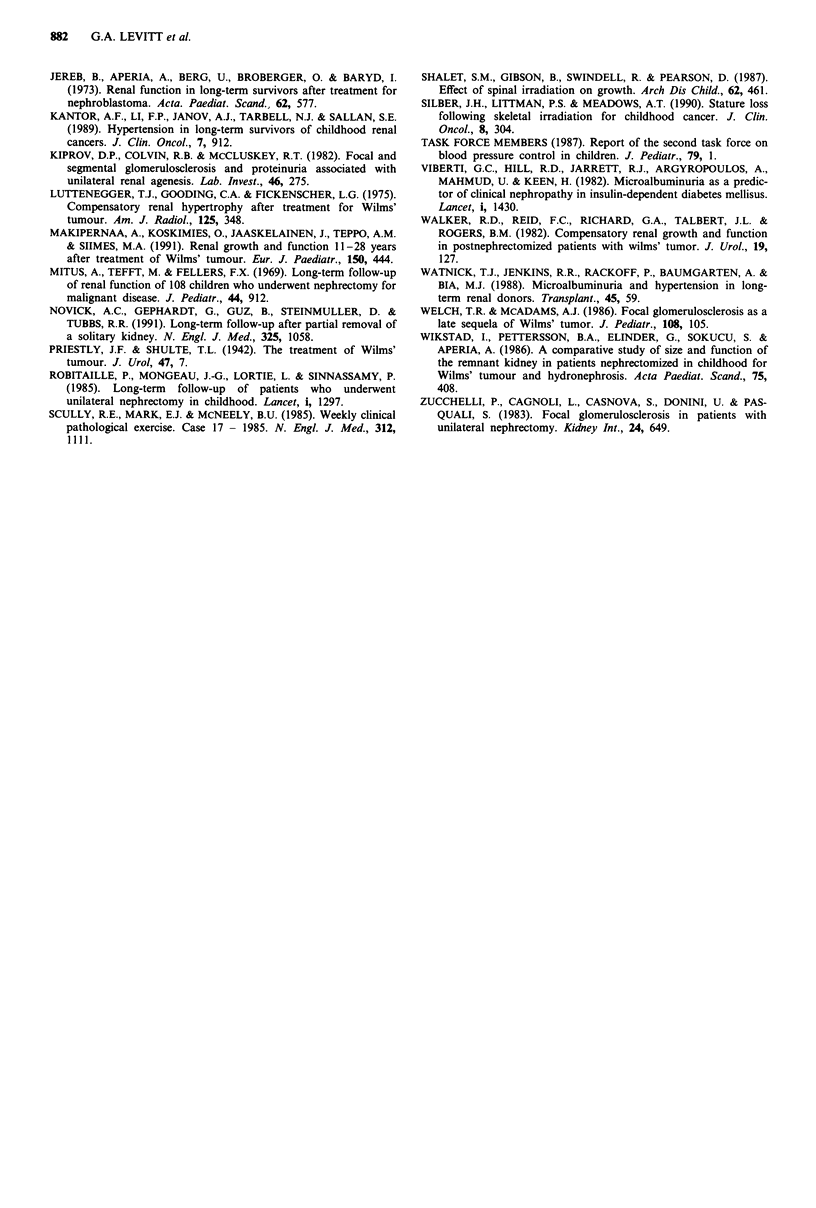# Renal size and function after cure of Wilms' tumour.

**DOI:** 10.1038/bjc.1992.378

**Published:** 1992-11

**Authors:** G. A. Levitt, E. Yeomans, C. Dicks Mireaux, F. Breatnach, J. Kingston, J. Pritchard

**Affiliations:** Department of Haematology, Hospital for Sick Children, London, UK.

## Abstract

Now that most patients with Wilms' tumour are cured, it is practicable to study the long-term morbidity of their treatment and use this information to reduce treatment sequelae in the future. In this study we evaluate the size and function of the remaining kidney in 53 survivors of Wilms' tumour with a mean off treatment follow-up of 13 years. There was evidence of renal dysfunction in 17 (32%), including ten (19%) with a low GFR (< 80 ml/min/1.73 m2SA), six (11%) with hypertension and five (9%) with increased urinary albumin excretion. Measurements of renal size showed 'good' renal compensatory hypertrophy in only 55% of patients. 'Good' refers to renal size of more than 2 s.d. above the mean renal length for children with two kidneys. There were no correlations between GFR, renal size, blood pressure, microalbuminuria or type of treatment. However, children less than 24 months at diagnosis and children receiving chemotherapy with radiation doses to remaining kidney of more than 1200 cGy had a worse renal prognosis. Patients whose Wilms' tumour is diagnosed in infancy should have careful long-term follow-up of renal function and size. Older patients may safely be followed up less often, unless their remaining kidney was received > 1200 cGy.


Br. J. Cancer (1992), 66, 877–882 © Macmillan Press Ltd., 1992

### Renal size and function after cure of Wilms' tumour

G.A. Levitt[1], E. Yeomans[1], C. Dicks Mireaux[1], F. Breatnach[1,*], J. Kingston[2] & J. Pritchard[1]

[1]Departments of Haematology, Oncology and Radiology, Hospital for Sick Children, London; [2]Department of Paediatric Oncology, St Bartholomew's Hospital, London.

**Summary** Now that most patients with Wilms' tumour are cured, it is practicable to study the long-term morbidity of their treatment and use this information to reduce treatment sequelae in the future. In this study we evaluate the size and function of the remaining kidney in 53 survivors of Wilms' tumour with a mean off treatment follow-up of 13 years. There was evidence of renal dysfunction in 17 (32%), including ten (19%) with a low GFR (<80 ml/min/1.73 m$^2$ SA), six (11%) with hypertension and five (9%) with increased urinary albumin excretion. Measurements of renal size showed 'good' renal compensatory hypertrophy in only 55% of patients. 'Good' refers to renal size of more than 2 s.d. above the mean renal length for children with two kidneys. There were no correlations between GFR, renal size, blood pressure, microalbuminuria or type of treatment. However, children less than 24 months at diagnosis and children receiving chemotherapy with radiation doses to remaining kidney of more than 1200 cGy had a worse renal prognosis.

Patients whose Wilms' tumour is diagnosed in infancy should have careful long-term follow-up of renal function and size. Older patients may safely be followed up less often, unless their remaining kidney was received >1200 cGy.

More than half a century after the introduction of nephrectomy for the treatment of unilateral Wilms' tumour (Priestly *et al.*, 1942), there is little published data concerning growth and function of the remaining kidney. Several studies have shown that there is compensatory renal growth in patients treated for Wilms' tumour with multimodality therapy (Walker *et al.*, 1982; Luttenegger *et al.*, 1975; Dinkel *et al.*, 1988), but less than in children who have nephrectomy for non-malignant conditions. There are few reports of both renal growth and function in long-term survivors of Wilms' tumour and the number of patients in these studies is small (Wikstad *et al.*, 1986; Makipernaa *et al.*, 1991).

The issue is of particular current interest because of recent reports of delayed morbidity in patients with renal agenesis or after nephrectomy for non-malignant conditions. Some of these patients have presented with hypertension and proteinuria due to focal glomerulosclerosis (Watnick *et al.*, 1988; Kiprov *et al.*, 1982), possibly the consequence of hyperfiltration-induced damage to the remaining glomeruli (Zucchelli *et al.*, 1983; Hostetter, 1984). There are also case reports describing glomerulosclerosis in long-term survivors of Wilms' tumour (Welch *et al.*, 1986; Scully *et al.*, 1985).

These findings, if substantiated, would have implication for the design of new treatment protocols and long-term follow-up strategies. The aim of our study was to measure renal growth and function in long-term survivors of unilateral Wilms' tumour and to identify risk factors for renal damage.

### Patient characteristics and methods

Eighty-two consecutive survivors of Wilms' tumour, Stage I–IV, diagnosed between 1970–1980 at the centres, Hospital for Sick Children and St. Bartholomew's Hospital, London, were eligible for the study which ran from August 1988 to April 1990. Fifty-four patients agreed to participate. One was later withdrawn from analysis as, 8 years after the diagnosis of a right sided Wilms' tumour, she needed surgical treatment for a left sided adrenal phaeochromocytoma which may have contributed to her renal dysfunction.

Twenty-six patients were female. The mean age at diagnosis was 3.4 ± 0.17 years (range 0.6–10.2), approximately the median age for Wilms' tumour at diagnosis (Green *et al.*, 1979). Mean follow-up time was 12.9 ± 3.0 years (range 7.8–19) with a mean age at time of study 16.1 ± 3.6 years (range 9.5–24.1). Twenty-eight were post-pubertal and two girls had babies of their own during the study period. One patient had hemihypertrophy but otherwise there were none of the congenital abnormalities sometimes seen in Wilms' tumour patients. Tumour stage and treatment are shown in Table I. Many had been treated in the Medical Research Council 1st and 2nd Wilms' Tumour Trials and three patients were in the 1st United Kingdom Childrens Cancer Study Group Wilms' Tumour Study.

Twenty-five patients (47%) had stage I disease. The treatment for Stage I disease varied over the period of the study: four patients had a nephrectomy only because they were aged less than 18 months at diagnosis. One child was treated with surgery and renal bed radiotherapy alone and nine with nephrectomy and chemotherapy (vincristine or actinomycin-D or both for 6–12 months) without irradiation. The remainder had both abdominal radiotherapy and chemotherapy. The 28 stage II–IV patients were all irradiated and received a combination of two, three, or four cytotoxic drugs (vincristine, actinomycin, ± doxorubicin ± cyclophosphamide) over 6–22 months. The radiation dose to the surviving kidney was dependant on the field and kidney shielding. By reviewing all simulator films and treatment sheets, the dose of radiation to the remaining kidney was calculated for each patient. Twenty-three patients received less than 1200 cGy (130–1200) and 17 children received doses ranging from 1200–1720 cGy.

The 28 patients not studied had a comparable median age at diagnosis of 3.3 years (range 0.5–12). The stage distribution were similar with 44% patients with stage I disease. More patients were in treatment Group I (30%) and Group III (48%) than the study group but detailed radiation doses were not calculated.

Each patient was admitted as a day case for the investigations. A full blood count, electrolytes, urea and creatinine were measured. Glomerular function was assessed in two ways. The glomerular filtration rate (GFR) was measured from the plasma clearance of 51-chromium diaminotetra-acetic acid ($^{51}$Cr EDTA) calculated from plasma samples at 2 and 4 h and corrected to 1.73 m$^2$ surface area (SA) (Chantler *et al.*, 1972). Microalbuminuria (Viberti *et al.*, 1982) was used as a sensitive measure of renal glomerular damage. An overnight urine specimen was obtained and the urine albumin

Correspondence: G.A. Levitt, Department of Haematology and Oncology, Hospital for Sick Children, Great Ormond Street, London WC1N 3JH, UK.

*Present address: Department of Paediatric Oncology, Our Lady's Hospital for Sick Children, Crumlin, Dublin 12.

Received 21 November 1991; and in revised form 15 June 1992.

**Table I** Study patients' stage and treatment $n = 53$

| Stage | No. | Nephrectomy | Nephrectomy chemotherapy | Nephrectomy chemotherapy radiotherapy 1 | Nephrectomy chemotherapy radiotherapy 2 | Nephrectomy radiotherapy 1 |
|---|---|---|---|---|---|---|
| I | 25 | 4 | 9 | 11 | 0 | 1 |
| II | 10 | – | – | 5 | 5 | – |
| III | 13 | – | – | 5 | 8 | – |
| IV | 5 | – | – | 1 | 4 | – |

Radiotherapy 1 = < 1200 cGy; Radiotherapy 2 = ≥ 1200 cGy.

concentration measured by radioimmunoassay and expressed as a ratio to urinary creatinine concentration (UA/UC) measured in mg mmol$^{-1}$. The values were compared with those from 77 normal children (Gibb, 1990). Albumin was measured using a commercially available kit with a sensitivity of 0.5 mg l$^{-1}$. The ratios were log transformed before statistical analysis; results were expressed as the geometric mean and range ( ± s.d.) calculated on logged data.

An aliquot of the overnight urine specimen from patients with microalbuminuria was tested for abnormalities reflecting renal tubular dysfunction. Retinol binding protein (RBP) is a low molecular weight protein freely filtered by the glomerulus and reabsorbed by the proximal tubule. It was measured in urine by an enzyme linked immunosorbent assay using rabbit antisera (Gibb, 1990) and expressed as a ratio to creatinine.

Blood pressure was measured on three occasions by two observers (EY, GL) with patients on bedrest. A random zero sphygmomanometer was used to reduce observer bias. The nearest two values were averaged and a standard deviation scores (SDS) calculated using Task Force on Blood Pressure Control in Children 1987 data matched for sex and age (Task Force Member, 1987). The mean blood pressure SDS were compared to zero using an unpaired $t$-test.

Plasma renin activity was measured in venous blood after the patient had 2 h bedrest by radioimmunoassay of angiotensin I generated (Dillon *et al.*, 1975). A midstream urine was obtained for urine analysis and culture.

Kidney size was measured using a Siemens SL2 ultrasound machine with a 3.5 or 5 megahertz probe. The patients were in the prone position and three readings were taken in each plane. The renal length standard deviation scores were calculated using Dinkel's data for renal length in healthy controls with two kidneys matched for kidney laterality and patient height (Dinkel *et al.*, 1985). Good renal compensatory hypertrophy was defined on kidneys measuring more than two standard deviation above the mean for the controls.

A similar study (Breatnach, F., unpublished) had been performed between 1980–1982 on 43 of the 53 children reported here. The method of GFR estimation was similar and therefore direct comparison was valid but methods of blood pressure estimation and renal size measurement were not considered comparable between the two studies. Parameters were compared with the $t$-test and correlations were investigated using linear regression anaylsis.

**Figure 1** Frequency histogram of glomerular filtration rates.

**Figure 2** Glomerular filtration rate 1st *vs* glomerular filtration rate 2nd study.

### Results

One patient had *E. coli* urinary tract infection on the study day; and her data were included in this analysis. Full blood count, electrolytes, urea and creatinine were normal in all patients.

### *Glomerular function*

The mean GFR estimated from the plasma clearance of $^{51}Cr$ EDTA was 89.9 ± 13.7 ml/min/1.73 m$^2$ SA (Figure 1). Ten patients had GFR < 80 ml/min/1.73 m$^2$ SA. Forty-three of the patients had, as described been studied between 1980–1982 and there was good correlation between the GFRs (Figure 2 $P < 0.001$), the mean GFR change was + 11.2 ml/min/1.73 m$^2$ SA, ( ± 11.3).

The geometric mean UA/UC for the Wilms' tumour patients was 0.45 (range 0.09–23.26), not significantly different from the mean of the controls 0.32 mg mol$^{-1}$ (range 0.05–1.95) (Viberti *et al.*, 1982). Increased albumin excretion was detected in five children (range 2.1–23.26 mg ml$^{-1}$). These five children, one of whom had developed diabetes mellitus six years before the study, were screened for tubular proteinuria (RBP) but no increase was detected.

### *Blood pressure and plasma renin activity*

Six children (11%) were either hypertensive (Systolic blood pressure SDS > 2.0) or on treatment for hypertension. The mean systolic blood pressure SDS excluding the two children who were on antihypertensive treatment was 0.84 ± 1.03

**Figure 3** Urinary albumin/urinary creatinine ratios in controls and study patients.

**Figure 4** Frequency histogram of systolic BP SDS.

(range −1.80 to +3.80) (Figure 4). The mean diastolic blood pressure SDS was 1.15 (±1.11) (range −1.4 to +4.3). Both systolic and diastolic BP SDS were significantly greater than zero ($t = 7.3$ $P < 0.001$). Of 51 children tested, plasma renin activity was abnormal in three children but one child, was considered not to have been on complete bedrest, and neither of the other two were hypertensive.

*Renal size*

Renal length ranged from −0.55 to 5.80 (Figure 5) with a mean renal length SDS of 2.19 ± 1.52 (range −0.55 to 5.80) (Figure 5). Renal length scores did not correlate with time since nephrectomy. Adequate post-nephrectomy compensatory renal hypertrophy (renal length >2 s.d.) was considered to have occurred in 29 children. In the other 24 there was 'inadequate' renal growth (<2 s.d.).

No overall correlation was found between renal length, GFR, microalbuminuria, systolic blood pressure or type and intensity of treatment.

**Figure 5** Frequency histogram of renal length SDS.

Children who received no radiation (13 patients Group I), children receiving radiation of <1200 cGy (23 patients Group II), and children receiving ⩾1200 cGy (17 patients Group III) were compared (Table II). There were no significant differences in blood pressure but in Groups I and III the mean GFR was significantly lower than in Group II ($P < 0.05$). Mean renal size in Group III was also significantly less than in Group II ($P < 0.05$). Group III had a significantly higher mean UA/UC value than the 77 normal controls ($P < 0.01$).

There was evidence of renal dysfunction in the children with inadequate renal size (<2 s.d.) compared with the remaining children. Significantly more children with small kidneys have lower GFR ($P < 0.02$) although the differences in the mean GFR are not significant (Table III).

The most significant finding, however, was age at time of nephrectomy ($P < 0.01$). Children less than 24 months old at time of surgery had poor subsequent renal growth (Figure 6) and in comparison with older children there was a trend to have low mean GFR and higher mean systolic BP SDS (Table IV). Yet these children have had less treatment for Wilms' tumour. In four, nephrectomy alone was performed and of the seven who received chemotherapy six received Group II treatment with only one child receiving 1200 cGy to the remaining kidney.

One child from Group II with a high GFR 123 ml/min/1.73 m$^2$ SA had microalbuminuria. This child was not hypertensive, but may be showing early signs of hyperfiltration syndrome.

### Discussion

These patients represented 67% of all the children presenting with unilateral Wilms' tumour to two specialist oncology centres between 1970–1980. There was no known selection bias, the refusal of patients or parents to be studied being the main cause for exclusion.

Stage I disease was diagnosed in 47% of patients, a higher proportion than would be expected at diagnosis, reflecting the better survival of these children (Walker *et al.*, 1982). This study is the largest to investigate both renal size and function in patients more than ten years from treatment. Seventeen (32%) of patients, with a mean follow-up period of 13 years had evidence of renal dysfunction, in addition 14 patients had only small kidneys. Two-thirds of all the patients had received less than 600 cGy to the remaining kidney.

Nine of the ten patients with low GFR had also been tested 8 years previously. All of them had a GFR<85 ml/min/1.73 m$^2$ SA at that time. Overall, the GFR has increaed between the two studies (Figure 2). The mean GFR of 90 ml/min/1.73 m$^2$ SA is in line with other studies after nephrectomy for non-malignant and malignant conditions

**Table II** Results in relation to treatment groups

| | *Group I* | *P value* | *Group II* | *P value* | *Group III* |
|---|---|---|---|---|---|
| Radiotherapy | None | | < 1200 cGy | | ⩾ 1200 cGy |
| No. | 13 | | 23 | | 17 |
| Mean renal length SDS (± s.d.) | 1.9 (± 1.8) | | 2.8 (± 1.3) | $P<0.05^{b}$ | 1.7 (± 1.5) |
| No. of patients SDS < 2 | 8 | | 7 | | 9 |
| Mean GFR ml/min/1.73 $m^2$ SA (± s.d.) | 86.5 (± 11.1) | $P<0.05^{a}$ | 95.0 (± 11.7) | $P<0.05^{b}$ | 84.5 (± 16) |
| No. of patients GFR < 80 ml/min/1.73 $m^2$ SA | 4 | | 2 | | 4 |
| Mean systolic BP SDS (± s.d.) | 1.1 (± 0.8) | | 0.9 (± 1.0) | | 0.8 (± 0.9) |
| No. of patients SDS > 2 or on treatment for hypertension | 1 | | 1 | | 4 |
| Geometric mean urinary albumin/ creatinine ratio mmol/mg (range) | 0.29 (0.1–1.21) | | 0.41 (0.09–7.26) | | 0.70 (0.11–23.26) |
| Abnormal urinary albumin/creatinine ratio | 0 | | 3 | | 2 |

[a]*P* value between Group I and II; [b]*P* value between Group II and III. No other significant differences were found.

**Table III** Results in relation to size of kidney

| Renal length | < 2 s.d. | > 2 s.d. | *P* value |
|---|---|---|---|
| No. of patients | 24 | 29 | |
| Mean GFR ml/min/1.73 $m^2$ SA (± s.d.) | 87 (± 13) | 93 (± 14) | NS |
| GFR < 80 ml/min/1.73 $m^2$ SA | 8 | 2 | < 0.02 |
| Mean systolic BP SDS (± s.d.) | 1.1 (± 0.9) | 0.8 (± 1.0) | NS |
| Systolic BP > 2 SDS or on treatment for hypertension | 4 | 2 | NS |
| Geometric mean urinary albumin/creatinine mmol/$mg^{-1}$ (range) | 0.47 (0.12–7.26) | 0.42 (0.09–23.26) | NS |
| Abnormal urinary albumin/creatinine ratio | 6 | 7 | NS |
| Age at nephrectomy < 24 months | 13 | 5 | < 0.01 |

**Figure 6** Renal length *vs* height.

(Walker *et al.*, 1982; Robitaille *et al.*, 1985) Ten (19%) patients had poor renal clearance and will need long term surveillance.

There has been controversy regarding the risk of a hyperfiltration syndrome resulting in a glomerulosclerosis (Kiprov *et al.*, 1982; Zucchelli *et al.*, 1983; Robitaille *et al.*, 1985; Novick *et al.*, 1991) in patients who have had nephrectomy. Novick *et al.* suggested proteinuria was inversely proportional to the remaining functional renal mass and patients with a reduction of more than 75% were at particular risk. Microalbuminuria is a sensitive index of glomerular damage. Five patients had increased protein excretion of glomerular origin and all of them had received irradiation (240–1704 cGy). One adolescent patient 10 years off treatment had increased his GFR by 24% in 8 years to 123 ml/min/$m^2$ SA (> 2 s.d. above the mean for the Wilms' cohort); although his kidney length was 2.2 SDS, he may be showing features of hyperfiltration. Of the remaining four patients, two had small kidneys and a low GFR and one child had diabetes mellitus, a known cause of increased protein excretion (Gibb *et al.*, 1989).

Six patients (11%) were hypertensive or on treatment for

**Table IV** Results in relation to age at nephrectomy

| Age at nephrectomy | <24/12 | >24/12 |
|---|---|---|
| No. of patients | 18 | 35 |
| Mean renal length SDS (± s.d.) | 1.7 (±1.3) | 2.4 (±1.6) |
| Renal length <2 s.d. | 13 | 11 |
| Mean GFR ml/min/1.73 $m^2$ SA (± s.d.) | 88.8 (±10.7) | 90.5 (±14.8) |
| GFR <80 ml/min/1.73 $m^2$ | 4 | 6 |
| Mean systolic BP SDS (± s.d.) | 1.13 (±0.75) | 0.98 (±0.84) |
| Systolic BP>2 s.d. or on treatment for hypertension | 2 | 4 |
| Geometric mean urinary albumin/ creatinine ratio mmol/mg (range) | 0.34 (0.1-3.2) | 0.51 (0.09-23.26) |
| Abnormal urinary albumin/creatinine ratio | 1 | 4 |

hypertension. Our cohort had elevated systolic and diastolic BP SDS. Significantly greater than zero ($t = 7.3$, $P<0.001$). Kantor *et al.* (1989) studied 119 adult survivors of childhood renal tumours and concluded that hypertension was not a complication of Wilms' tumour or its treatment. However, there was no standardisation of the techniques used for blood pressure measurements in their study.

Ultrasound was used to measure renal length because it is non-invasive, requires no contrast medium and has minimum inter-observer error. Earlier studies all used intravenous pyelograms and comparison of kidney size with $L_1-L_5$ length, an inaccurate measurement in patients receiving radiotherapy. In normal children renal length is shown to correlate with height (Dinkel *et al.*, 1985) and so, to evaluate our data on children investigated at differing ages, we calculated a renal length SDS from published data on height and renal length in normal children. This method is also obviously flawed because 40 children received radiation to the lumbar spine and four also had thoracic spine irradiation for pulmonary metastases. Shalet *et al.* (1987) studied the effect of radiation on spinal growth of patients receiving craniospinal radiation and calculated a maximum loss of 9 cm. The value was dependent on age at time of radiation. Silber *et al.* (1990) developed an equation to calculate height loss which took into account dose, age at the time of radiation and length of spine involved. Using the equation our patients' estimated height loss was 6-8 cm i.e. approximately 3% of their height which we felt would only marginally overestimate their renal length SDS. Poor compensatory renal growth (<2 s.d.) was associated with low GFR and may be a useful non-invasive follow-up screening test to identify patients at risk.

The various treatment modalities appear not to influence late morbidity. Patients receiving chemotherapy and renal bed or hemiabdominal radiation appear to fare better than the children who receive no radiation or whole abdominal radiation. Other workers (Walker *et al.*, 1982; Luttenegger *et al.*, 1975; Jereb *et al.*, 1973) have found no relationship between intensity of treatment and GFR or renal size. The no radiation group (I) consists mostly (Zucchelli *et al.*, 1983; Green *et al.*, 1979) of children <24 months at diagnosis and these young children have significantly smaller kidneys and sometimes have other forms of nephropathy. Wilms' tumour and genito-urinary abnormalities have been associated with nephropathy in the form of DRASH syndrome (Jadresic *et al.*, 1990). It could be speculated that children with Wilms' tumour presenting early in life may have a predisposition to glomerular pathology linked to chromosomal deletions near the Wilms' gene/s. Mitus (Mitus *et al.*, 1969) in her study of 108 patients, mentions four Wilms' survivors who had no radiation of whom three had low GFR with two of the three receiving no chemotherapy. Their ages were not stated but one wonders whether they were young at diagnosis. There have also been anecdotal reports (Welch *et al.*, 1986; Scully *et al.*, 1985) of patients less than 24 months at diagnosis presenting with glomerulosclerosis at more than 10 years from diagnosis. It is difficult to determine whether these patients presented as a result of hyperperfusion or because of pre-existing glomerular disease. Our data maybe suggests the latter.

In summary, renal well-being cannot be guaranteed after cure from Wilms' tumour. Around one-third of our patients had evidence of renal impairment, apparently associated with young age at diagnosis and radiation >1200 cGy to the remaining kidney. Larger intercentre studies are needed to clarify these findings but, for the time being at least long-term follow-up of cured Wilms' patients is mandatory. Histological studies of the residual kidney tissue surrounding the Wilms' tumour, may be particularly revealing. Partial nephrectomy and abandonment of whole abdominal radiation may reduce renal morbidity in future generations.

G.A.L. is supported by the Leukaemia Research Fund and J.P. is supported, in part, by the Imperial Cancer Research Fund.

We would like to thank Dr R. Redpath née Sandland for measuring the radiation dose to the kidney and Professor T.M. Barratt for his help and encouragement throughout the study. Mrs V. Shah helped in analysing the samples. Mrs V. Spencer and Mr P. Reeve provided assistance in computing and statistical analysis. Thanks to Lisa Luxon and Denise Van Mol for typing the manuscript.

### References

CHANTLER, C. & BARRATT, T.M. (1972). Estimation of glomerular filtration rate from plasma clearance of 51-chromium edetic acid. *Arch. Dis. Child.*, **47**, 613.

DILLON, M.J. & RYNESS, J. (1975). Measurement of plasma renin activity by semi-micro radioimmunoassay of generated angiotensin. *I. J. Clin. Pathol.*, **28**, 625.

DINKEL, E., ERTEL, M., DITTRICH, M., PETERS, H., BERRES, M. & SCHULTE-WISSERMANN, H. (1985). Kidney sizes in childhood sonographical growth charts for kidney length and volume. *Pediatr. Radiol.*, **15**, 38.

DINKEL, E., BRITSCHO, J., DITTRICH, M., SCHULTE-WISSERMANN, H. & ERTEL, M. (1988). Renal growth in patients nephrectomized for Wilms tumour as compared to renal agenesis. *Eur. J. Pediatr.*, **147**, 54.

GIBB, D.M., TOMLINSON, P.A., DALTON, N.R., TURNER, C., SHAH, V. & BARRATT, T.M. (1989). Renal tubular proteinuria and microalbuminuria in diabetic patients. *Arch. Dis. Child.*, **64**, 129.

GIBB, D.M. (1990). *Early markers of the renal complications of insulin dependent diabetes mellitus in children.* MD Thesis, University of Bristol, 1990.

GREEN, D.M. & JAFFE, N. (1979). The role of chemotherapy in the treatment of Wilms' tumor. *Cancer*, **44**, 52.

HOSTETTER, T.H. (1984). The hyperfiltrating glomerulus. *Med. Clin. NA*, **68**, 387.

JADRESIC, J., LEAKE, J., GORDON, I. & 5 others (1990). Clinicopathologic review of twelve children with nephropathy, Wilms' tumour and genital abnormalities (DRASH Syndrome). *J. Pediatr.*, **117**, 717.

JEREB, B., APERIA, A., BERG, U., BROBERGER, O. & BARYD, I. (1973). Renal function in long-term survivors after treatment for nephroblastoma. *Acta. Paediat. Scand.,* **62,** 577.

KANTOR, A.F., LI, F.P., JANOV, A.J., TARBELL, N.J. & SALLAN, S.E. (1989). Hypertension in long-term survivors of childhood renal cancers. *J. Clin. Oncol.*, **7,** 912.

KIPROV, D.P., COLVIN, R.B. & MCCLUSKEY, R.T. (1982). Focal and segmental glomerulosclerosis and proteinuria associated with unilateral renal agenesis. *Lab. Invest.*, **46,** 275.

LUTTENEGGER, T.J., GOODING, C.A. & FICKENSCHER, L.G. (1975). Compensatory renal hypertrophy after treatment for Wilms' tumour. *Am. J. Radiol.*, **125,** 348.

MAKIPERNAA, A., KOSKIMIES, O., JAASKELAINEN, J., TEPPO, A.M. & SIIMES, M.A. (1991). Renal growth and function 11–28 years after treatment of Wilms' tumour. *Eur. J. Paediatr.*, **150,** 444.

MITUS, A., TEFFT, M. & FELLERS, F.X. (1969). Long-term follow-up of renal function of 108 children who underwent nephrectomy for malignant disease. *J. Pediatr.*, **44,** 912.

NOVICK, A.C., GEPHARDT, G., GUZ, B., STEINMULLER, D. & TUBBS, R.R. (1991). Long-term follow-up after partial removal of a solitary kidney. *N. Engl. J. Med.*, **325,** 1058.

PRIESTLY, J.F. & SHULTE, T.L. (1942). The treatment of Wilms' tumour. *J. Urol*, **47,** 7.

ROBITAILLE, P., MONGEAU, J.-G., LORTIE, L. & SINNASSAMY, P. (1985). Long-term follow-up of patients who underwent unilateral nephrectomy in childhood. *Lancet*, **i,** 1297.

SCULLY, R.E., MARK, E.J. & MCNEELY, B.U. (1985). Weekly clinical pathological exercise. Case 17 – 1985. *N. Engl. J. Med.*, **312,** 1111.

SHALET, S.M., GIBSON, B., SWINDELL, R. & PEARSON, D. (1987). Effect of spinal irradiation on growth. *Arch Dis Child.*, **62,** 461.

SILBER, J.H., LITTMAN, P.S. & MEADOWS, A.T. (1990). Stature loss following skeletal irradiation for childhood cancer. *J. Clin. Oncol.*, **8,** 304.

TASK FORCE MEMBERS (1987). Report of the second task force on blood pressure control in children. *J. Pediatr.*, **79,** 1.

VIBERTI, G.C., HILL, R.D., JARRETT, R.J., ARGYROPOULOS, A., MAHMUD, U. & KEEN, H. (1982). Microalbuminuria as a predictor of clinical nephropathy in insulin-dependent diabetes mellisus. *Lancet*, **i,** 1430.

WALKER, R.D., REID, F.C., RICHARD, G.A., TALBERT, J.L. & ROGERS, B.M. (1982). Compensatory renal growth and function in postnephrectomized patients with wilms' tumor. *J. Urol.*, **19,** 127.

WATNICK, T.J., JENKINS, R.R., RACKOFF, P., BAUMGARTEN, A. & BIA, M.J. (1988). Microalbuminuria and hypertension in long-term renal donors. *Transplant.*, **45,** 59.

WELCH, T.R. & MCADAMS, A.J. (1986). Focal glomerulosclerosis as a late sequela of Wilms' tumor. *J. Pediatr.*, **108,** 105.

WIKSTAD, I., PETTERSSON, B.A., ELINDER, G., SOKUCU, S. & APERIA, A. (1986). A comparative study of size and function of the remnant kidney in patients nephrectomized in childhood for Wilms' tumour and hydronephrosis. *Acta Paediat. Scand.*, **75,** 408.

ZUCCHELLI, P., CAGNOLI, L., CASNOVA, S., DONINI, U. & PASQUALI, S. (1983). Focal glomerulosclerosis in patients with unilateral nephrectomy. *Kidney Int.*, **24,** 649.